# A Molecularly Modulated Mode-Locked Laser

**DOI:** 10.1038/s41598-018-30743-9

**Published:** 2018-08-15

**Authors:** Shin-ichi Zaitsu, Takao Tsuchiya

**Affiliations:** 1 Department of Applied Chemistry, Graduate School of Engineering, Kyushu University, 744 Motooka, Nishi-ku, Fukuoka, 819-0395 Japan; 2 Division of International Strategy, Center for Future Chemistry, Kyushu University, 744 Motooka, Nishi-ku, Fukuoka, 819-0395 Japan

## Abstract

A mode-locked laser operating at a frequency over 10 THz is reported, which is three orders of magnitude greater than a standard mode-locked laser. The system used molecules with a Raman gain as an amplifier, while coherent molecular motions were used for optical modulation. Molecules in a high-finesse optical cavity modulated a continuous-wave beam to produce a train of ultrashort optical pulses at a repetition rate corresponding to the frequency of molecular motion. Phase-locking was achieved by an appropriate compensation of the total dispersion of the optical cavity. Thus, the oscillating multiple longitudinal modes were all coupled under phase-matching conditions of parametric four-wave mixing.

## Introduction

Mode-locked lasers are powerful tools for the exploration of ultrafast phenomena in physical systems, as well as for producing high-intensity electromagnetic radiation fields^1,2^. State-of-the-art systems can produce, directly from the laser oscillator, pulses of just a few electromagnetic wave cycles at bandwidths over one-octave^3^. The output pulse energy has been dramatically increased to several tens of *μ*J at tens of MHz repetition rates in the sub-picosecond region^4^. Average output powers directly from the oscillator that are greater than the kilowatt level have been reported^5^. The combination of high peak intensities and average powers is achieved by coherent stacking of the ultrashort pulses in an external enhancement cavity^6^. This enables extension of the wavelength range to the extreme ultraviolet via generation of higher-order harmonics^7^. The progress of ultrafast science and its applications in material processing, frequency metrology, and nonlinear optics relies on the continuing development of mode-locked laser technology.

An important aspect of a mode-locked laser is that of an oscillator having a well-defined frequency. Careful control of the repetition rate led to the invention of the optical frequency comb, which bridges ultrafast and precision spectroscopy. The repetition rate is an essential parameter in spectroscopy, metrology, and attosecond science^8–10^. Therefore, improvements in rate stability as well as increases in the rates are of great interest. At present, the highest reported repetition rate for a mode-locked pulse train from a single optical cavity was in the 100 GHz regime^11,12^. The demand for higher rates is rapidly growing in electron beam generation^13^, ultrahigh-capacity communications^14,15^, and optical sampling/clocks in all-optical data processing^15,16^.

However, further increases in the repetition rate are challenging because of the geometrical configuration of mode-locked lasers; i.e., the repetition rate is inversely proportional to the length of the optical cavity. Theoretically, a 10-THz mode-locked pulse train would be emitted from a 30-*μ*m optical cavity. To achieve rates greater than 1 THz, several approaches have been proposed, such as harmonic mode-locking in semiconductor lasers or distributed Bragg reflector-structured lasers^17,18^, nonlinear modulation in optical fibers^19^, and phase-locking between multiple continuous lasers^20^. However, the bandwidth limits further increases in repetition rate, and new approaches are needed to reach 10 THz. Cavity-enhanced four-wave mixing based on a continuous-wave laser is a promising way to overcome this limitation^21^. However, the longitudinal modes contributing to the mode-lock operation were limited to three lines^22^.

Here, an approach is described that produces a 10 THz rate by using coherent molecular motion as an optical modulator and the Raman gain of molecules as an amplifier. The mode-locked optical pulses are generated by the interaction of a fundamental continuous-wave (cw) beam with molecules in a dispersion-compensated optical cavity. The use of molecular *orth*-hydrogen for modulation and amplification led to the generation of a 17.6-THz ultrafast optical pulse train that corresponds to the rotational frequency of the molecules, and is 10^3^ larger than that of common mode-locked lasers.

**Figure 1:**
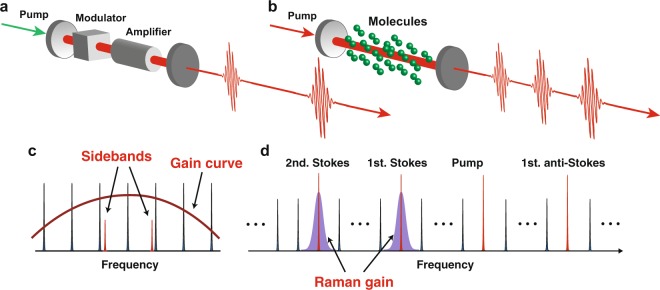
Basic concept of a molecularly modulated mode-locked laser. (**a**) Configuration of a typical mode-locked laser. (**b**) Configuration of a molecularly modulated mode-locked laser. (**c**) Relative relationships between longitudinal modes of an optical cavity, a gain curve provided by an amplifier, and sidebands arising from intracavity optical modulation. (**d**) Longitudinal modes contributing to the operation of a molecularly modulated mode-locked laser. Raman gains provided by intracavity stimulated Raman scattering overlap with longitudinal modes in the optical cavity.

Factors that determine the repetition rate of a mode-locked laser can be understood in terms of the frequency domain, as shown schematically in Fig. 1. Figure 1a is the basic configuration of a mode-locked laser consisting of four main components: an optical cavity, an amplification medium, an optical modulator, and a dispersion compensator. Its operational frequency, i.e., the repetition rate of emitted optical pulses *ν*, is determined by the free spectral range of the optical cavity, which is, in turn, determined by the optical cavity length *l* as follows:

1 $$\nu =c\mathrm{/2}l,$$

where *c* is the speed of light. When the laser system is pumped by an external power source, the longitudinal modes within the gain bandwidth of the amplifier oscillate. If the frequency of the intracavity optical modulator exactly matches the free spectral range of the cavity, all the oscillating modes become highly correlated because the sidebands arising from the optical modulation have a definite phase relationship (see Fig. 1c). This situation enables formation of a train of optical pulses by Fourier synthesis in the time domain, and the basic operation leads to two essential factors that determine the repetition rate: (1) the length of the cavity and (2) the frequency of the intracavity modulator.

An increase in the repetition rate requires a decreased cavity length and an increased frequency of the intracavity modulator. An addition problem is that optical modulators based on the electro-optic effect do not operate effectively in the terahertz range. A mode-locked laser with an extremely short cavity length and an extremely high modulation frequency has yet to be reported. An alternative approach to overcome these limitations uses gaseous, Raman-active molecules (see Fig. 1b). Coherent molecular motions serve as an intracavity optical modulator, and the Raman gain amplifies the longitudinal modes of the cavity. The approach enables ultrafast modulation and an extended cavity length at the same time. Macroscopically, an ensemble of coherent molecular motion can be regarded as an oscillation of the optical polarizability at the frequency of molecular motion^23^. The interaction between the coherent molecular motion and the light produces phase-locked sidebands in the same way as does an electro-optic modulator. Since frequencies of molecular motions (vibration or rotation) are generally greater than the THz range, molecular optical modulation (MOM) could operate at frequencies not accessible (~GHz) to conventional electro-optic and crystalline optical modulators^24^. Here, a MOM would be operating at more than 10 THz. In addition, the intracavity molecules are used as an amplification medium because they have Raman gains at separation frequencies corresponding to the modulation frequency in the longer wavelength region (see Fig. 1d). If the cavity length is such that the free spectral range is larger than the bandwidth of the Raman gain, then only one mode in the gain curve can oscillate. The phase-locked sidebands generated via MOM get amplified, producing multiple single-frequency modes separated by the frequency of the molecular motion, even when the cavity length is not sufficiently short for operation at terahertz frequencies. Although the sidebands in the shorter wavelength region are not amplified by the Raman gain, they evolve under the phase-matched conditions for parametric four-wave mixing (FWM). This occurs for an appropriate compensation of the cavity dispersion^21^, accompanied by the growth of the sidebands in the longer wavelength region. Because all the oscillating modes at the shorter and longer wavelength sides of the pump frequency are mutually phase-locked due MOM, the system works as “a molecularly modulated mode-locked laser”.

**Figure 2:**
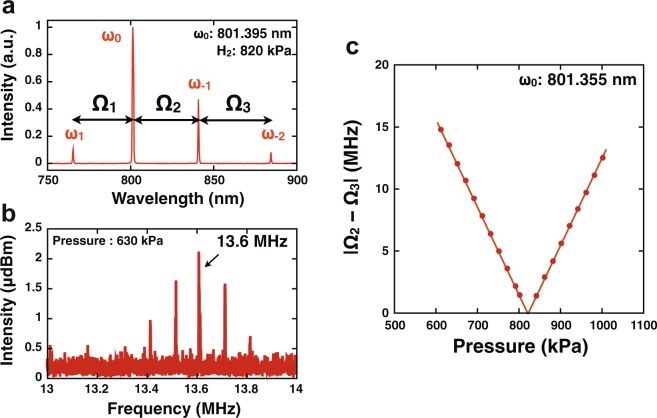
(**a**) Output spectrum of a molecularly modulated mode-locked laser. (**b**) Spectrum from nonlinear optical detection of a beam from the laser. (**c**) Intracavity hydrogen pressure dependence of the frequency of the signal from the nonlinear optical detector. The frequency corresponds to the frequency difference between |Ω_2_ − Ω_3_| shown in (**a**).

To find the mode-locked condition, the pump beam wavelength and the intracavity hydrogen pressure were controlled to enable oscillation of four emission lines at a uniformly spaced frequency interval. This is because the concentration of molecules filled in the optical cavity directly determines the total cavity dispersion that is critical for the mode-locked operation, and it can be assumed to be proportional to the intracavity pressure in the range we used in this experiment. The *ω*_0_ pump beam was coupled with an optical cavity filled with molecular hydrogen (see supplemental information for details). Figure 2a is a spectrum of the four emission lines, *ω*_1_, *ω*_0_, *ω*_−1_, and *ω*_−2_, separated by the interval of 17.6 THz that corresponds to the frequency of *ortho*-hydrogen rotational motion. In this case, *ω*_0_ was 801.395 nm and the intracavity hydrogen pressure was 820 kPa. *ω*_−1_, and *ω*_−2_ were amplified via Raman gains at the expense of the fundamental *ω*_0_, *ω*_1_ was generated through Raman-resonant FWM under phase-matched conditions^21^. Ω_1_ = *ω*_1_ − *ω*_0_ and Ω_2_ = *ω*_0_ − *ω*_−1_ (see Fig. 2a) were equal because of energy conservation in parametric FWM between *ω*_0_, *ω*_−1_, and *ω*_1_. Whereas, Ω_3_ = *ω*_−1_ − *ω*_−2_ = Ω_2_ was not given because *ω*_−2_ was attributed to stimulated Raman scattering (SRS), which is an inelastic process accompanied by a change in kinetic energy^25^. To find a pressure to satisfy Ω_3_ = Ω_2_, the signal from a nonlinear detector was monitored as a function of the intracavity hydrogen pressure (see Methods for details). A typical spectrum using the detector is shown in Fig. 2b, and the dependence of the frequency on hydrogen pressure is shown in Fig. 2c. The hydrogen pressure that satisfied Ω_3_ = Ω_2_ was 821 kPa, which also enabled the oscillation of the four phase-locked emission lines to generate a 17.6-THz train of ultrashort optical pulses.

**Figure 3:**
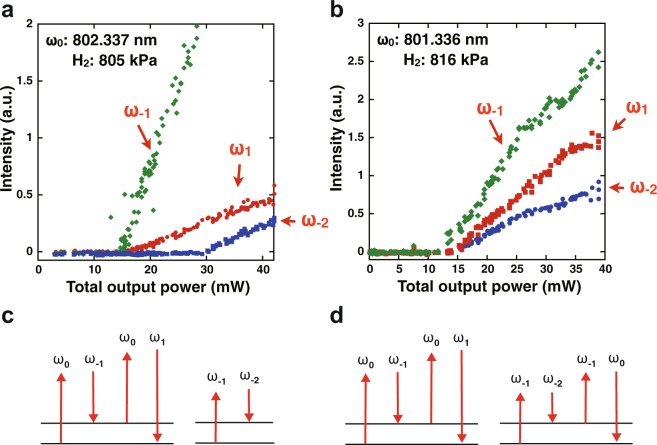
Evolution of intensities of *ω*_1_, *ω*_−1_, and *ω*_−2_ in the output beam as a function of the total beam power. (**a**) Different thresholds for *ω*_−1_ and *ω*_−2_. (**b**) The threshold of *ω*_−1_ correlates with that of *ω*_−2_ at a 816-kPa hydrogen pressure and at *ω*_0_ of 801.366 nm. (**c**) Energy diagram when *ω*_−2_ is generated through stimulated Raman scattering. (**d**) Energy diagram when *ω*_−2_ is generated through four-wave mixing under the phase-matched condition.

There was a coincidence of the thresholds for Raman line generation at the pressure that enabled the mode-locked emission discussed above. Figure 3a,b plot all the Raman line intensities as a function of the total output power at hydrogen pressures of 805 kPa and 816 kPa, respectively. Figure 3a shows the behavior observed at a wide range of intracavity hydrogen pressures; *ω*_1_ and *ω*_−1_ together generated a total output power of 15 mW, which was below the 30-mW threshold for *ω*_−2_ emission. The coincidence of the *ω*_1_ and *ω*_−1_ thresholds was attributed to the simultaneous generation of photons at *ω*_1_ and *ω*_−1_ via phase-matched FWM at the expense of two photons at *ω*_0_ (see Fig. 3c). Whereas, a photon at *ω*_−2_ was provided by SRS at a threshold determined by the intracavity power of *ω*_−1_. The difference in the generation processes for *ω*_−1_ and *ω*_−2_ led to a threshold gap between *ω*_−1_ and *ω*_−2_. In contrast to Fig. 3a, the 816-kPa data in Fig. 3b indicated that the generation of *ω*_2_ was coincident with that of *ω*_0_ and *ω*_−1_. These results strongly imply that phase-matched FWM, instead of SRS, contributed to the generation of *ω*_−2_ (see also Fig. 3d). Hence, all four emission lines were coupled via FWM, satisfying three phase-matching conditions: Δ*k* = 2*k*_0_ − *k*_1_ − *k*_−2_ = 2*k*_−1_ − *k*_−2_ − *k*_0_ = *k*_−1_ + *k*_−2_ − *k*_1_ − *k*_0_ = 0. This was only allowed when the four emission lines were separated by a constant frequency interval. The coincidence of the thresholds for *ω*_−1_ and *ω*_−2_, and *ω*_1_ indicated that mode-locking of the multi-frequency emission lines occurred at 801.3 nm and a 816-kPa hydrogen pressure. Mode-locked operation required precise control of the total intracavity dispersion for intracavity FWM phase-matching.

**Figure 4:**
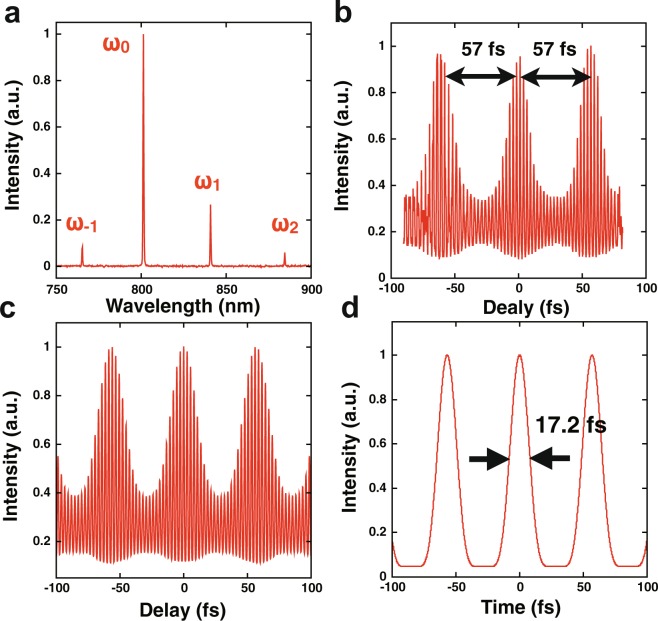
(**a**) A spectrum of the beam used for the measurement of the autocorrelation trace shown in (**b**). (**b**) Autocorrelation trace of a train of optical pulses from the molecularly modulated mode-locked laser. The interval between adjacent pulses was a constant 57 fs. (**c**) Autocorrelation trace calculated using the spectrum shown in (**a**) assuming a constant spectral phase. (**d**) Temporal waveform of a train of optical pulses calculated from the Fourier synthesis of the spectrum in (**a**). The full-width at half-maximum of each optical pulse was 17.2 fs and the repetition rate was 17.6 THz.

The spectrum and the interferometric autocorrelation trace of the output beam observed under the conditions described above are shown in Fig. 4a and b, respectively. Because the frequency interval between the four emission lines was 17.6 THz, the repetition rate of the optical pulses was 17.6 THz in the time domain. The autocorrelation trace in Fig. 4b had a 57-fs periodic structure that was the reciprocal inverse of 17.6 THz. Figure 4c,d show a calculated autocorrelation trace and a temporal waveform, respectively, from an electric field given by the Fourier synthesis of the spectrum in Fig. 4a, assuming a constant spectral phase. The trace in Fig. 4c agrees well with the measured one in Fig. 4b, which strongly suggests that a 17.6-THz train of ultrashort pulses with 17.2-fs full widths at half maximum (Fig. 4d) was emitted from the molecularly mode-locked laser.

It should be noted that the stability of the pulse train was not experimentally verified. The key parameter for verification would be phase coherence in the multi-frequency emission lines. Phase coherence has been reported for three emission lines arising from molecular modulation of a cw beam^26^. The phase coherence between the four emission lines here should be measured to better understand the proposed molecular-modulated mode-locked laser. The generation of a train of ultrashort pulses at a repetition rate greater than 10 THz could be supported by the following results: (1) mutual coupling between the four emission lines under a specific condition described above, and (2) good agreement between calculated and measured autocorrelation traces in Fig. 4a,c, respectively. In addition, it is noteworthy to mention about the control of a repetition rate of this laser system. The repetition rate is determined by the Raman shift frequency of a hydrogen molecule, i.e. 17.6 THz for S_0_(1) transition of *ortho*-hydrogen. It is stable against change of temperature because the Raman shift frequency of gaseous hydrogen has no significant dependence on temperature. However, the Raman shift frequency has a bandwidth due to Doppler broadening^27^, e.g. ~1 GHz at a pressure of 0.1 MPa at room temperature, and it depends on temperature and density. The repetition rate of the laser system can be modified within this bandwidth by varying the separation frequency between the longitudinal modes of the laser cavity.

A mode-locked laser has been demonstrated that operates via optical modulation by coherent molecular motions and amplification by molecular Raman gain. A train of ultrashort pulses was produced at a repetition rate greater than 10 THz, which is three orders of magnitude larger than that of a standard mode-locked laser. A hydrogen-filled, 10-cm optical cavity with a pair of negative dispersion mirrors enabled compensation of the total intracavity dispersion over a frequency range greater than 50 THz. The optical cavity was pumped by a single-frequency, near-infrared cw laser, and the output beam had four emission lines separated by a uniform interval of 17.6 THz, which corresponded to a *ortho*-hydrogen rotational frequency. Phase-locking at the constant frequency interval between the emission lines was achieved when the total intracavity dispersion was correctly compensated, and was verified by nonlinear optical detection of line frequency deviations. The coincidence in the thresholds of the multi-frequency emission indicated coupling between the emission lines under the phase-locked condition. Interferometric autocorrelation indicated good agreement with that calculated under the assumption of constant phase in the spectrum. Overall, these results strongly suggest generation of a train of 17.2-fs pulses at a repetition rate of 17.6 THz. This will be an important step in the development mode-locked lasers with considerably increased operational frequencies.

## Methods

### Nonlinear detection of frequency mismatch between multi-frequency emission lines

Nonlinear optical detection was used to evaluate phase-locking of the molecularly modulated mode-locked laser by qualitative measurements of deviations from the equally separated frequencies of three emission lines^28^. For the three emission lines at *ω*_−1_, *ω*_0_, and *ω*_1_, the frequency separations are: Ω = *ω*_0_ − *ω*_−1_ and Ω′ = *ω*_1_ − *ω*_0_. If the phases of the emission lines are expressed as *ϕ*_*n*_(*t*) = *ω*_*n*_*t* + *ϕ*_*n*0_ for *n* = −1, 0, 1, where *ϕ*_*n*0_ is the initial phase for each emission line, phase-locking requires that the following conditions be fulfilled at any given time:

$${\varphi }_{0}(t)-{\varphi }_{-1}(t)={\varphi }_{1}(t)-{\varphi }_{0}(t\mathrm{).}$$

In the frequency domain, the separations between adjacent lines are identical:

$${\rm{\Delta }}{\rm{\Omega }}={\rm{\Omega }}-{\rm{\Omega }}^{\prime} =0.$$

Thus, ΔΩ = 0 ensures mode-locked operation with a pulse train at a repetition rate of Ω. Furthermore, deviation from the phase-locked state of a molecularly modulated mode-locked laser can be determined quantitatively by the measurable parameter, ΔΩ. Here, the three modes are expressed as *E*_−1_ = exp [−*i*(*ω*_0_ − Ω)*t*]; *E*_0_ = exp [−*iω*_0_*t*]; and, *E*_1_ = exp [−*i*(*ω*_0_ + Ω + ΔΩ)*t*]. A waveform synthesized from these lines is expressed as *E*(*t*) = *E*_−1_ + *E*_0_ + *E*_1_. When *E*(*t*) is measured with a detector that provides a signal proportional to the square of the intensity of an input beam, the output signal is:

$${I}_{2}(t)={[E(t)E{(t)}^{\ast }]}^{2}=15+4\,\cos \,{\rm{\Delta }}{\rm{\Omega }}t+16\,\cos \,{\rm{\Omega }}t+16\,\cos ({\rm{\Omega }}+{\rm{\Delta }}{\rm{\Omega }})t+\cdots .$$

In the above equation, the terms with frequencies larger than 2Ω are omitted. The second term with frequency ΔΩ can be measured with a nonlinear detector having a commercially available bandwidth. Furthermore, this term is independent of the frequency of the mode separation Ω. This allows discrimination between phase-locked and non-phase-locked states. The nonlinear detector was a photomultiplier tube (PMT, Hamamatsu 1P28). Its Sb-Cs photocathode had a two-photon response in the near-infrared region (>650 nm), as evidenced by the quadratic output voltage dependence on the 800-nm laser beam emission, even at power levels <1 mW (see ref.^28^ for details). A spectrum analyzer (Advantest, U3741) was used to measure the frequency components at ΔΩ in the PMT signal.

### Estimation of a condition for mode-locked operation

Control of the total dispersion in the optical cavity was essential for mode-locked operation of the system emitting phase-locked multi-frequency lines separated by a constant frequency interval. The cavity mirrors had a negative group delay dispersion over the entire region, with high reflectivity. (The dispersive property of the cavity mirrors are shown in the supplementary information in ref.^24^). To estimate the frequency intervals between the oscillating modes, it was assumed that the four emission lines, *ω*_0_, *ω*_1_, *ω*_−1_, and *ω*_−2_, were oscillating in the hydrogen-filled optical cavity pumped at 790–810 nm. *ω*_0_ was the frequency of the pump beam, *ω*_0_ and *ω*_−2_ were Stokes emission lines, and *ω*_1_ was the single anti-Stokes line. Frequency differences between these emission lines (Ω_j_, j = 1, 2, 3) were calculated by the following equation using a group refractive index *n*_g_ for hydrogen, and a *ϕ*_m_ group delay for one bounce on the cavity mirror.

$${{\rm{\Omega }}}_{{\rm{j}}}=\sum _{{\rm{N}}={{\rm{N}}}_{0}}^{{{\rm{N}}}_{{\rm{j}}}}\,\frac{c}{2(L{n}_{{\rm{g}}}(\omega )+c{\varphi }_{{\rm{m}}}(\omega ))}$$

*ω*_N_ was the frequency of the Nth longitudinal mode and N_j_ was the number of the longitudinal mode responsible for *ω*_j_, j = −1, 0, 1, 2. Mode-locking was achieved when Ω_1_ = Ω_2_ = Ω_3_ was satisfied at a 738-kPa hydrogen pressure and a pump wavelength of (*ω*_0_) of ~797.5 nm. Multi-frequency emission under these conditions enabled generation of an optical pulse train that had a repetition rate equal to the 17.6-THz mode interval.

## Electronic supplementary material

Supplementary Information
