# Supplementary material for: A Molecularly Modulated Mode-Locked Laser
Source: Sci Rep. 2018 Aug 15;8:12175. doi: 10.1038/s41598-018-30743-9 (PMC6093895; doi:10.1038/s41598-018-30743-9)
Supplement: Supplementary file 1 — Supplementary Information [file 41598_2018_30743_MOESM1_ESM.pdf]

A Molecularly Modulated Mode-Locked Laser

Shin-ichi Zaitsu[1,2], Takao Tsuchiya[1]

[1]*Department of Applied Chemistry, Graduate School of Engineering, Kyushu University, 744 Motooka, Nishi-ku, Fukuoka 819-0395, Japan*

[2]*Division of International Strategy, Center for Future Chemistry, Kyushu University, 744 Motooka, Nishi-ku, Fukuoka 819-0395, Japan*

SUPPLEMENTARY INFORMATION

1 Experimental setup

The molecularly modulated mode-locked laser used molecules filled in an optical cavity pumped by a continuous-wave (cw) laser to excite molecular coherence and stimulated Raman gain. Specifically, a single-frequency Ti:sapphire laser (Coherent Inc., MBR-110) operating at near-infrared wavelengths was used as the pump beam, which was coupled into the optical cavity through a beam expander focusing lens to match it with the lowest transverse mode of the optical cavity. The Fabry-Perot type optical cavity was $\sim$80-mm in length and had a pair of plano-concave mirrors with 250-mm curvatures. The cavity was installed in a stainless-steel chamber equipped with input and output windows for beam passage. The chamber was filled with Raman-active molecules for optical modulation and amplification; the intracavity pressure was less than 1 MPa. The spectrum of the output beam was acquired with a fiber-coupled multichannel spectrometer. Deviation from

phase-locking between the multi-frequency components was measured via nonlinear optical detection, as described in the Methods section of the main text. The temporal characteristics of the output beam were measured with an interferometric autocorrelator equipped with a photomultiplier that had the same- photocathode as that in the nonlinear optical detector. The autocorrelator was also equipped with a pair of beam splitters that were coated on one side and placed in a back-to-back configuration to eliminate asymmetric dispersion contributions from the beam splitter substrates. This enabled measurement of the temporal waveform of the output beam, including multiple-frequency components over a broad range (750-900 nm).

2 Design of the optical cavity

The molecularly modulated mode-locked laser required an optical cavity that allowed the oscillation of equally spaced multiple longitudinal modes separated by frequencies greater than 10 THz. Molecular hydrogen was used for the modulation medium as well as the gain medium. The frequency of a rotational Raman transition of *ortho*-hydrogen at a room temperature ($S_0(1)$: $J = 1 \rightarrow J = 3$) was 17.6 THz. Hence, the modulation frequency, i.e., the repetition rate of the mode-locked laser, was also 17.6 THz. A broadband high-finesse optical cavity was required to generate the multi-frequency emission lines via intracavity cw Raman lasing based on the molecular Raman gain[1]. We used a pair of cavity mirrors having a reflectance of $\sim$99.97% and a transmittance of $\sim$0.02% from 740 nm to 925 nm, which covers all the oscillating wavelengths in this experiment. The bandwidth of the Raman gain curve of the $S_0(1)$ transition depended on the hydrogen pressure; it was less than 1 GHz at a pressure of several hundred kPa[2]. The free spectral

range (FSR) of the optical cavity had to be larger than the Raman gain bandwidth to overlap a single longitudinal mode with the Raman gain curve. To satisfy this condition, the length of the optical cavity was 8 cm (FSR: $\sim$1.8 GHz). Figure 1d in the main text is a schematic of the longitudinal modes of the optical cavity and the Raman gain curves. The latter appeared at an interval of 17.6 THz for the $S_0(1)$ transition of $ortho$-hydrogen; the high-finesse region of the optical cavity was large enough to include the five emission lines that were equally separated by 17.6 THz.

References

1. Zaitsu, S. & Imasaka, T. Continuous-wave multifrequency laser emission generated through stimulated Raman scattering and four-wave Raman mixing in an optical cavity. *IEEE J. Quantum Electron.* **47,** 1129-1135 (2011).

2. Herring, G. C., Dyer, M. J. & Bischel, W. K. Temperature and density dependence of the linewidths and line shifts of the rotational Raman lines in N_2 and H_2. *Phys. Rev. A* **34,** 1944-1951 (1986).
